# Impact of Street Medicine on Emergency Department Use: A Three-Year Evaluation of People Experiencing Homelessness

**DOI:** 10.5811/westjem.63074

**Published:** 2026-09-09

**Authors:** Savannah Vetterly, Amy Wozniak, Theresa Nguyen

**Affiliations:** * Loyola University of Chicago, Stritch School of Medicine, Maywood, Illinois; † Loyola University of Chicago, Clinical Research Office, Maywood, Illinois; ‡ Loyola University Medical Center, Loyola Center for Community & Global Health, Department of Emergency Medicine, Maywood, Illinois

## Abstract

**Introduction:**

People experiencing homelessness (PEH) frequently utilize the emergency department (ED) for acute and primary healthcare needs. Loyola Street Medicine is a multidisciplinary team comprised of physicians, students, and other professionals who provide weekly healthcare services to PEH at a train station in Chicago. The objective of the study was to identify the specific patient demographics, chief complaints, and clinical interventions associated with increased odds of these patients reporting that they would have sought care at an ED had Loyola Street Medicine been unavailable.

**Methods:**

We conducted a retrospective review of patients seen by Loyola Street Medicine between September 2021 and September 2024, with 2,121 total encounters and a subset of 1,565 with identifying data. The primary outcome measure was whether the patient reported they would have gone to the ED for care, and the secondary outcome measures were patient demographics, chief complaints, and interventions provided. We estimated odds ratios with univariable logistic regression mixed models, with random intercept accounting for multiple patient visits in the subset of patients with identifiers.

**Results:**

For 63% of all encounters (*N* = 1,293), people experiencing homelessness indicated they would have gone to the ED without street medicine, with the most common demographics including Black race (77%), male sex (80%), and a mean age of 46.7 years. Among patients who indicated they would have gone to the ED, the most prevalent chief complaints were wound check (22%), musculoskeletal issues (17%), and podiatry concerns (14%). Black and “other” race patients had decreased odds of indicating they would have gone to the ED compared to White patients (0.69 [95% CI, 0.49–0.97], *P* = .03 and 0.39 [95% CI, 0.22–0.69], *P* = .001, respectively). Patients with wound-related complaints and those needing wound care as an intervention had greater odds of indicating they would go to the ED versus any other location such as a clinic or a primary care physician (1.46 [95% CI, 1.07–1.99], *P* = .02 and 1.5 [95% CI, 1.11–2.03], *P* = .009, respectively).

**Conclusion:**

The findings demonstrate that street medicine can play a vital role in addressing the complex needs of people experiencing homelessness. By providing consistent, low-barrier care, street medicine has the potential to reduce nonemergent ED use and improve continuity of care.

## INTRODUCTION

At a given point in time, an estimated 771,480 people experience homelessness (PEH) in the United States.^1^ Homelessness presents a significant public health challenge that compounds chronic disease management and complicates acute care delivery. People experiencing homelessness have an estimated average life expectancy of 42 to 52 years.^2^ Barriers to healthcare for this population include lack of insurance, high costs, transportation limitations, stigma, and an overall lack of resources, resulting in lower primary care use.^3^,^4^ While primary care is underused among PEH, the emergency department (ED) is frequently overused and serves as the setting that these patients rely on for care.^4^

### Emergency Department Use

Between 2016 and 2021, there were approximately 1,441,000 ED visits made annually by unhoused individuals in the US.^5^ They were seen an average of 5.7 times per year at the same ED compared to 3.2 times for housed patients and had significantly longer lengths of stay (LOS).^6^ Further, PEH were over seven times more likely to return to the ED within the first 30 days from the prior visit and approximately 11 times more likely within the next two years compared to housed individuals with Medicaid.^7^ Patients who are discharged from the ED to a shelter or the streets are also at an increased risk of readmission, further complicating their care and use of healthcare resources.^8^

Their more frequent use of the ED places a strain on the healthcare system and contributes to significant costs. Admissions for PEH have been estimated to cost $2,559 more than admissions for housed patient after adjusting for demographic factors and intensity of resource use.^9^ People experiencing homelessness are admitted with a higher frequency and tend to have longer hospital LOS, according to a national dataset analysis.^6^ Among PEH who are Medicaid/Medicare eligible in Southern California, 87% of total costs were attributable to the ED, hospital, and use of skilled nursing facility use.^10^

People experiencing homelessness are also at higher risk of trauma and violence. Emergency department visits from these patients were often related to injury at higher rates than housed patients (47.5% versus 33.8%, respectively) across the US.^6^ Their use of the ED is further complicated by the trimorbidity of homelessness, which is defined by the overlap of mental health issues, substance use disorder, and chronic medical conditions. A national study showed increased odds of alcohol and substance use disorder-related diagnoses in PEH presenting to the ED.^6^ In Boston, behavioral health disorders accounted for about one third of ED visits and one half of hospitalizations among PEH.^11^ Qualitative interviews with ED staff and people experiencing housing insecurity emphasized that a key factor in improving their healthcare and decreasing ED use is better overall communication and connection with community resources.^12^

### Street Medicine

Street medicine is an innovative approach to delivering healthcare by meeting patients where they are physically located, whether it be in an encampment, a shelter, under bridges, or another unsheltered environment.^13^ A typical street medicine team consists of an interdisciplinary group of physicians, nurses, students, and outreach workers who provide medical care and address the social needs of PEH. While there is variation between street medicine teams in terms of care capabilities, most teams manage medications for chronic conditions, provide wound care, address acute complaints, and provide referrals to community resources. There has also been a more recent focus on incorporating psychiatric care. One study found that an outreach team for PEH had an average decrease of approximately 2.3 predicted ED visits per person in the 12-month follow-up period compared to the pre-enrollment period.^14^ However, studies are limited on the intersection between street medicine and ED use.

**Population Health Research Capsule**

What do we already know about this issue?

*Street medicine may reduce emergency department (ED) use among persons experiencing homelessness (PEH)*.

What was the research question?

*What factors are linked to greater odds of PEH reporting they would have used the ED without street medicine?*

What was the major finding of the study?

*63% of PEH stated they would have gone to the ED without street medicine. Wound care was the most common reason (22%)*.

How does this improve population health?

*Our study demonstrates that street medicine may reduce ED use while improving continuity of care and healthcare access for PEH*.

The number of street medicine teams has grown in the US in recent years. Loyola Street Medicine was established in January 2020 in response to the unmet medical needs of Chicago’s unsheltered homeless population. On a given day, there are an estimated 7,452 PEH, 1,316 of whom are unsheltered, according to the 2025 Chicago Point-in-Time (PIT) Count.^15^ Every Thursday from 8 pm to midnight, volunteers from Loyola Street Medicine have provided medical care at the Chicago Transit Authority Blue Line train station since September of 2021. On a given week, the typical outreach team consists of 6–10 volunteers, which includes a board-certified emergency physician or internal medicine physician who supervises all patient encounters, medical students, and nursing students. Psychiatrists, dentists, and ophthalmologists also volunteer, as do residents from a variety of specialties.

Most patient encounters take 10 to 20 minutes depending on the number of complaints, the complexity of their medical issues, and the length of time the patient is willing to stay. The typical number of patients seen per weekly outreach is 20–35, a number that continues to grow. The Chicago Transit Authority train station was chosen to maximize convenience for individuals who were already using the train as their primary shelter, based on the 2020 PIT count. In fact, 43.2% of people experiencing unsheltered homelessness in the city reported that they would spend the night on buses and trains.^16^

### Present Study

While street medicine has been demonstrated to decrease ED use among PEH, it is usually discussed within a conceptual framework or from a program rather than patient level.^12^,^17^ Limited research has shown a reduction in the number of ED visits after patients engage in street medicine with outreach services.^14^ However, a gap remains in the literature that specifically asks PEH where they would have sought care and whether they would have gone to the ED without street medicine. While the demographics, presenting complaints, and interventions associated with their ED utilization have been studied,^6^,^18^ to our knowledge no prior research has investigated the demographics, presenting complaints, and interventions required among PEH who reported they would have sought care in the ED without street medicine.

## METHODS

This retrospective observational study aimed to describe PEH who reported they would have gone to the ED for care, as well as to identify patient demographics, chief complaints, and interventions associated with patients indicating self-reported intent of ED use without street medicine. For each week of service between September 13, 2021, and September 26, 2024, data were collected for each patient encounter. Recorded variables included patient initials, date of birth, age, sex, race, length of homelessness, insurance, chief complaint, intervention provided, and response to the question, “Where would you go for care if Loyola Street Medicine was not here tonight?” This information was logged by the student, physician, or other health professional who was primarily involved in the encounter with the patient.

A single abstractor performed all chart coding using a standardized data collection tool (REDCap) with predefined variable definitions. Records were then regularly reviewed with the principal investigator to ensure consistency in coding. Worster et al (2005) describe the criteria for the adherence to methodologic standards in medical record review studies; the present study adhered to the following criteria: abstractors training; variable definition; use of a data abstraction form; medical record identified; abstractors’ performance monitored; missing-data management plan; and institutional review board (IRB) approval.^19^ This study was approved by the IRB at Loyola University of Chicago (LU#218244). Based on our research questions, the primary outcome measure was whether the patient stated that they intended to go to the ED for care, and the secondary outcome measures were patient demographics, chief complaints, and intervention provided.

### Chief Complaint

After each encounter, the patient’s chief complaint was recorded. There was no limit on the number of chief complaints, and each chief complaint was uniquely coded. However, if there were two complaints within the same category, this was coded only once (eg, if blood pressure check and glucose check were a patient’s chief complaints, this was coded once as a wellness check). After assessing all chief complaints, the following categories were created: Respiratory; Musculoskeletal (MSK); Trauma/Injury; Medication Refill; Wound Check; Dermatology/Bites; Wellness Check; Ear Nose Throat (ENT)/Upper Respiratory Infection (URI)/Allergy; Gastrointestinal (GI); Dental Pain; Vaccines; Psychiatric; Neurology; Podiatry; Ophthalmology; Substance use; Genitourinary (GU); Cardiology/Chest Pain; Frostbite; and other. If a patient had a foot wound or foot pain, it was coded as podiatry rather than wound check or MSK, respectively.

### Intervention

The following categories were created based on the most common interventions provided by the Loyola Street Medicine team: medication administered; vaccine administered; wound care; MSK intervention; referral to ED or 911 call; referral to social work; referral to primary care physician; foot care; and vitals/physical exam. Patients were not limited to one intervention for the purposes of coding, and similarly to the chief complaint category, two interventions in the same category were coded only once. For the medication administered, this was further subcategorized into the following: over-the-counter medications; inhalers; antibiotics or prescription medications; topicals; eye drops; prescription refill; and other. If a vaccine was administered, it was further subcategorized as influenza or COVID-19 vaccine.

### Research Question 1: Where would you go for care if Loyola Street Medicine was not here tonight?

Each patient was asked the above question and given the following options: ED; primary care physician (PCP); clinic; would not have pursued care; does not know where to go; or other. It is important to note that this outcome represented a self-reported hypothetical measure of intended healthcare use. Because patients ultimately engaged with the street medicine team, there was no mechanism to verify whether and when they might have ultimately presented to the ED or other location. If a patient listed a particular physician’s name, this was coded as PCP. If a particular hospital was listed, this was coded as ED. The specific response options for this question were mutually agreed upon with our community partner, the Night Ministry, which was also tracking this question during their street outreach.

### Statistical Methods

There were 2,121 patient encounters between September 2021 and September 2024. For all encounters, we summarized categorical values with counts and percentages, and we summarized continuous variables with means and standard deviations. For the full anonymized data, no formal hypothesis tests were performed, and descriptive statistics were provided for the first research question. Between September 13, 2021, and September 29, 2022 (classified as “year 1”), the patient’s date of birth was not collected, as it was not initially included among the predefined variables on the data collection instrument. Of the 2,121 total encounters, there were 1,613 observations in years 2 and 3 when date of birth was collected. Of those 1,613 encounters, 1,565 had date of birth and “patient initials” data, which represented 706 unique patients (see supplemental materials Figure 1 for a flow diagram). The 1,565 patient encounters for which there were identifiers comprised the subset group analyzed for the second research question. For this subset of the total encounters, we estimated odds ratios (OR) and corresponding 95% confidence limits with univariable logistic regression mixed models, with random intercept accounting for multiple patient visits. This study followed a single group of patients with no controls for which descriptive statistics were calculated; a subset of the total patient encounters with identifiers was used to estimate OR with univariable logistic regression mixed models.

### Research Question 1

The first research question describes the demographics, chief complaints, and interventions among all 2,121 patient encounters (ie, those patients who indicated they would have otherwise gone to the ED. As all encounters were used for this research question (even those without identifiers), all data are descriptive given that no adjustment for patient correlations could be performed.

Of all encounters, 1,293 patients (63%) indicated that if they had not sought care with Loyola Street Medicine, they would have gone to the ED. The demographics of these patients included Black race (77%), male sex (80%), and a mean age of 46.7 years (Table 1); 48% of those indicating potential ED visits had Medicaid insurance and 23% did not have insurance. Of those who indicated possible ED use, wound check (22%), musculoskeletal (17%), and podiatry (14%) were the most common chief complaints (Table 2).

As shown in Table 1, there was a higher percentage of individuals who were homeless between 1–5 years among PEH who would have sought care in the ED (36%) compared to those who indicated they would have sought other care (29%). Additionally, there was a higher percentage of PEH presenting with wound-related complaints who indicated they would seek care in the ED (22%) versus those who would have gone elsewhere (16%; Table 2). Among PEH who indicated they would have sought care in the ED, there was a lower percentage of wellness check as the chief complaint (7%) compared to those who indicated they would not have gone to the ED (12%). These percentages are for descriptive purposes only, as no statistical comparisons were performed for research question 1. Table 3 includes the interventions provided by Loyola Street Medicine stratified by whether PEH would have otherwise gone to the ED versus another location (including PEH who would not have sought care).

### Research Question 2: How likely is it that you would use the emergency department if it were not for street medicine?

The second research question aimed to identify demographics, chief complaints, and interventions associated with an increased odds of PEH indicating they would have gone to the ED versus any other location. To answer this question, a subset of the total patient encounters with identifiers with initials and date of birth was used for analysis (N = 1,565). We used univariable logistic regression mixed models with random intercept accounting for multiple visits to calculate OR and corresponding 95% confidence intervals. Two-sided *P* values < 0.05 were deemed statistically significant. All analyses were performed with SAS 9.4 (Cary, NC).

Black individuals and those with “other” self-identified race had lower odds of reporting they would have gone to the ED compared to White individuals as shown in Table 4 (0.69 [95% CI, 0.49–0.97], *P* = .03 and 0.39 [95% CI, 0.22–0.69], *P* = .001, respectively). Patients with “other” homelessness status (eg, intermittent, unknown, or homeless but unsure for how long) also had lower odds of reporting they would have gone to the ED than those experiencing homelessness for <1 year (0.69 [95% CI, 0.49–0.97], *P* = .03). Those with wound checks as a chief complaint had greater odds of indicating they would go to the ED versus any other location as shown in Table 5 (1.46 [95% CI, 1.07–1.99], *P* = .02). Similarly, as depicted in Table 6, patients needing wound care as an intervention had greater odds of indicating they would have gone to the ED versus any other location (1.5 [95% CI, 1.11–2.03], *P* = .009). If 911 was called or an ED referral was made, there were greater odds of the patient expressing they would have gone to the ED versus any other location (3.02, [95% CI, 1.35–6.74], *P* = .007).

## DISCUSSION

The findings of the study demonstrate that street medicine can be a crucial component of care for PEH. Further, 63% of the patients seen at the CTA Blue Line Station stated that they would have otherwise gone to the ED for care. While the results should not be interpreted as the actual percentage of individuals who would have otherwise gone to the ED, given that this measure was self-reported, the results do suggest that many PEH would consider going to the ED if it were not for street medicine. Further, this study characterizes the potential chief complaints that could have been seen in the ED if it were not for street medicine, with wound care, musculoskeletal, and podiatry complaints being the most frequent among individuals who indicated they would have otherwise gone to the ED. Only 4% of PEH who stated that they would have gone to the ED for care were subsequently referred to the ED after being assessed by Loyola Street Medicine, which demonstrates the ability for street medicine to manage a variety of medical issues and chief complaints that might have otherwise resulted in ED use.

Research has largely focused on conceptually arguing for the need for street medicine or relies on qualitative data.^10^,^17^ This relative lack of quantitative data extends to the connection between street medicine and potential ED deferral. In a survey of 25 California-based street medicine teams, a recurring theme was that street medicine allows for continuity of care compared to the typical pattern of ED use.^20^ Editorials have also described its potential to save the healthcare system significant amounts of money through reduced ED use but provide specific program level reports and more hypothetical data for cost savings.^10^ While concept papers have highlighted the importance of street medicine as a cost-effective means to decrease ED use, they do not provide original quantitative data.^21^ In a scoping review of 15 articles on street medicine and mobile clinics, no studies examined where individuals would have gone for care without street medicine; the current evidence of decreased ED use is heterogeneous across outcome definitions.^17^ Further, Lynch et al found that “SM [street medicine] studies have lacked methodological rigor…”^10^

While scoping reviews and landscape surveys identify street medicine as a means for ED deferral, the few quantitative studies that have demonstrated that street medicine has the potential to decrease ED visits significantly differ in methodology. For example, a study of 54 PEH found a reduction of 2.3 ED visits in the 12-month follow-up period with a street outreach team compared to the 12-month pre-enrollment period.^14^ However, without a control group, it is difficult to know whether the reduction was solely attributed to the outreach team. Based on review of current literature, most studies demonstrating that street medicine reduces ED visits are at the program level, examine a reduction in ED visits for their patients after engagement with street medicine. We use a longitudinal approach at the patient-level; to our knowledge, this is the only study that tracks individuals who did not go to the ED for care and instead engaged with street medicine services. No other studies have specifically asked patients where they would have gone for care (such as the ED) without street medicine.

Among the PEH population, individuals identifying as Black and “other” race had decreased odds of indicating they would have gone to the ED compared to White individuals. Studies that examine ED use among PEH emphasize that Black men are overrepresented in the ED, but this is often compared to the general population or non-PEH visits.^6^,^18^ Few studies have examined the demographics of homeless patients presenting to the ED as compared to those of the larger homeless population, and none have examined this in the context of street medicine. One Canadian study found that within a sample of homeless adults, Black participants had a lower rate of ED use but a higher rate of visits to social service professionals.^22^

In the United States, the majority of PEH are White men. However, Black men are overrepresented in the count relative to the number who identify as Black in the United States (32% versus 12%).^1^ A longstanding history of mistrust in the healthcare system could contribute to Black PEH having decreased odds of indicating that they would go to the ED if it were not for street medicine services.^23^ However, other studies not specific to homeless status have shown that Black patients are more likely than White patients to use the ED and less likely to use primary care.^24^,^25^ Further studies are needed to better understand the reasons for these ED trends, specifically for PEH.

Wound check was the most frequent chief complaint among this patient population. Wound care needs are common among PEH, with trauma wounds, lower leg ulcers, and frostbite wounds the most common.^26^ This is not surprising given the living conditions and environmental exposures that increase injury and infection risk within this population. Those with wound check as a chief complaint had significantly greater odds of indicating they would go to the ED, comprising 22% of potential ED visits without the presence of street medicine. In a nationally representative sample of PEH in the United States presenting to the ED, 55% of visits were due to injuries.^27^ However, studies examining ED visits by PEH often do not separate the chief complaints of injuries from wounds, making it difficult to assess the number of individuals going to the ED specifically for wound care. The statistically increased odds of indicating potential ED use among those with wound care complaints presenting to Loyola Street Medicine could mean that individuals have a greater concern for their wounds compared to other health problems. We posit that some PEH may not seek care for initially minor wounds until they get to a point that warrants a physician’s care. The study results highlight the importance of wound care training for street medicine physicians, students, and other professionals.

The findings from our specific study demonstrate that many PEH indicate that they would have gone to the ED for care if it were not for street medicine. Given that the average cost of an ED visit would be more expensive and use more resources than street medicine services, street medicine can be considered a more cost-effective solution. Startup funds for street medicine programs range between $5,000-$10,000, depending on departmental or university funding. The average cost of annual maintenance is $10,000–$15,000, but this can also vary widely depending on the breadth of services provided by the street medicine team. The cost of maintenance of Loyola Street Medicine is about $15,000 annually, with funding from the university, grants, and donations. Since there is no method to confirm where individuals would have sought care without street medicine, any specific estimate of the potential cost savings to our EDs and healthcare system would not be feasible. However, given that just 4% of the PEH who indicated they would have gone to the ED were actually referred to the ED after being assessed by Loyola Street Medicine, we conclude that street medicine can address many medical needs outside the acute setting of the ED.

Future studies could directly compare the chief complaints of PEH who present to the ED versus those who use street medicine services. Understanding whether there is a significant difference between these groups would better clarify the clinical needs that are met by both. Since PEH are transient and difficult to track, a multicenter study involving all street medicine teams in the city would better characterize ED use. As a follow-up study, it would also be interesting to assess how soon the individuals would have gone to the ED if it were not for street medicine. Further, while wound care was the most frequent complaint among those who stated they would have gone to the ED, it would be interesting to stratify the findings based on the type of wound (eg, gunshot wound, abscess). Future studies should examine the demographics and chief complaints of PEH who would have otherwise sought care at a primary care clinic or who would not have gotten any care.

The present study is one of the largest street medicine studies in the United States, including 2,121 patient encounters. While existing studies have examined healthcare use and access to services in the context of street medicine, this is the first to document where patients indicated they would have gone in the absence of street medicine and to categorize the chief complaints and interventions that could have potentially taken place in the local ED. Another strength of the study is that it includes longitudinal data over the span of three years, whereas other studies were primarily cross-sectional. Finally, the 20 categories of chief complaints, allowing for a more nuanced understanding of the conditions that PEH presented with who indicated they would have otherwise gone to the ED.

## LIMITATIONS

The study is not without its limitations. The study coincided with the COVID-19 pandemic, which could have impacted the prevalence of various chief complaints (eg, respiratory issues) or interventions (eg, vaccine administration) compared to typical years. Another limitation is the lack of standardization between individuals recording the chief complaint and interventions. Although all encounters were supervised by a physician or other healthcare professional, chief complaints were often documented by medical trainees, which likely introduced variability in both the number and type of complaints recorded across encounters. Another limitation concerns the question, “Where would you go for care if Loyola Street Medicine was not here tonight?” Some PEH named a local hospital, which was assumed to be the ED and was coded as such. A further limitation with this question is that it is self-reported, and therefore there is no feasible method to confirm definitive data on ED use, and the results should not be interpreted as such. In other words, while 63% of patients indicated they would have gone to the ED without street medicine, there is no way to confirm the exact amount of ED reduction or the exact number of each chief complaint through concrete data such as ED records. Finally, the results reflect the intersection between Loyola Street Medicine and the ED, which may not be representative of all street medicine groups.

## CONCLUSION

Our study demonstrates that street medicine and the emergency department are intertwined, with street medicine serving as a critical point of intervention and providing greater continuity of care for PEH. The findings are based on patient self-report, with many PEH stating that they would have gone to the ED without street medicine. The high prevalence of wound care-related complaints underscores the importance of proper wound care training to mitigate life-threatening wound care complications. Further research is needed to understand ED use patterns based on specific demographic factors such as age, race, and sex to better inform targeted interventions for this population.

## Supplementary Information



## Figures and Tables

**Table 1:** Demographics stratified by whether patients would have otherwise gone to the emergency department. (ED) without Loyola Street Medicine for all patient encounters between September 2021 and September 2024 in a study examining the intersection of street medicine and the ED.

Demographics	All data, *N* = 2,121	Not ED, *n* = 828	ED, *n* = 1,293
Age, mean (SD)	46.9 (11.9)	47.2 (11.7)	46.7 (12)
Male (%)	1,663 (79)	634 (77)	1,029 (80)
Race (%)			
White	370 (18)	128 (16)	242 (20)
Black	1,549 (77)	598 (77)	951 (77)
Other	100 (5)	55 (7)	45 (3)
Hispanic (%)	122 (6)	59 (7)	63 (5)
Insurance (%)			
Yes, but unknown	105 (5)	42 (6)	63 (5)
Medicaid	907 (47)	335 (46)	572 (48)
Medicare	59 (3)	21 (3)	38 (3)
Private	312 (16)	110 (15)	202 (17)
None	463 (24)	184 (25)	279 (23)
Other	69 (4)	31 (4)	38 (3)
VA	7 (0.5)	2 (0.3)	4 (0.4)
Unsure / unknown	14 (0.7)	10 (1)	4 (0.3)
Duration of homelessness (%)			
< 1 month (30 days)	53 (3)	22 (3)	31 (3)
1 month-6 months,	244 (13)	99 (15)	145 (13)
> 6 months-1 year	277 (15)	102 (15)	175 (15)
>1–5 years	606 (33)	197 (29)	409 (36)
> 5–10 years	162 (9)	61 (9)	101 (9)
> 10 years	122 (7)	46 (7)	76 (7)
Not homeless	206 (11)	90 (13)	116 (10)
Intermittent	18 (1)	5 (1)	13 (1)
Unknown	39 (2)	16 (2)	23 (2)
Homeless but unsure how long	91 (5)	30 (4)	61 (5)

Missingness: age, 34; sex, 7; race, 102; ethnicity, 24; insurance, 185; duration of homelessness, 303.

*ED*, emergency department; *SD*, standard deviation; *VA*, Veterans Affairs.

**Table 2:** Chief complaints stratified by whether patients would have otherwise gone to the emergency department (ED) without Loyola Street Medicine for all patient encounters between September 2021 and September 2024 in a study examining the intersection of street medicine and the ED.

Chief Complaint	All data, *N* = 2,121	Not ED, *n* = 828	ED, *n* = 1,293
Respiratory (%)	224 (11)	83 (10)	141 (10)
Musculoskeletal (%)	354 (17)	129 (16)	225 (17)
Trauma / Injury (%)	34 (2)	16 (2)	18 (1)
Medication refill (%)	165 (8)	67 (8)	98 (8)
Wound care (%)	418 (20)	129 (16)	290 (22)
Rash / bug bites / dermatology (%)	196 (9)	76 (9)	120 (9)
Wellness check (%)	195 (9)	99 (12)	96 (7)
ENT, URI, Allergy (%)	197 (9)	80 (10)	117 (9)
Abdominal pain / GI (%)	51 (2)	21 (3)	30 (2)
Dental pain (%)	43 (2)	15 (2)	28 (2)
Vaccines (%)	47 (2)	24 (3)	23 (2)
Psychiatric (%)	18 (1)	12 (1)	6 (0.5)
Neurology (%)	105 (5)	36 (4)	69 (5)
Podiatry (%)	300 (14)	116 (14)	184 (14)
Ophthalmology (%)	31 (1)	12 (1)	19 (1)
Complaints related to substance use (%)	31 (1)	11 (1)	20 (1)
GU (%)	58 (3)	21 (3)	37 (3)
Heart / Chest Pain (%)	23 (1)	8 (1)	15 (1)
Frostbite (%)	6 (0.3)	3 (0.4)	3 (0.2)
Other (%)	23 (1)	11 (1)	12 (1)
Number of Complaints (%)			
0	28 (1)	14 (2)	14 (1)
1	1713 (81)	672 (81)	1041 (81)
2	340 (16)	131 (16)	209 (16)
3 or 4	40 (2)	11 (1)	29 (2)

No missing data. A complaint was either mentioned, or it was considered absent.

*ED*, emergency department; *ENT*, ear, nose, and throat; *URI*, upper respiratory infection; *GI*, gastrointestinal; *GU*, genitourinary.

**Table 3:** Interventions stratified by whether patients would have otherwise gone to the emergency department (ED) without Loyola Street Medicine for all patient encounters between September 2021 and September 2024 in a study examining the intersection of street medicine and the ED.

Intervention	All data, *N* = 2,121	Not ED, *n* = 828	ED, *n* = 1,293
Medication administered (%)	1428 (67)	554 (67)	874 (68)
Type of medication administered (%)			
Over the counter	797 (38)	311 (38)	486 (38)
Inhaler	320 (15)	118 (14)	202 (16)
Antibiotics or prescriptions medicagtions	290 (14)	108 (13)	182 (14)
Topicals	349 (16)	113 (16)	216 (17)
Eye drops	19 (1)	9 (1)	10 (1)
Pharmacy / Prescription refill	23 (1)	8 (1)	15 (1)
Other	27 (1)	10 (1)	17 (1)
Vaccine administered? (%)	54 (3)	25 (3)	29 (2)
Type of vaccine (%)			
Influenza	16 (1)	7 (1)	9 (1)
COVID-19	46 (2)	22 (3)	24 (2)
Wound care (%)	447 (21)	137 (17)	310 (24)
MSK intervention (%)	91 (4)	41 (5)	50 (4)
Referred to ED (or 911 call) (%)	62 (3)	11 (1)	51 (4)
Referred to social work (%)	29 (1)	17 (2)	12 (1)
Referred to PCP (%)	71 (3)	29 (4)	42 (3)
Foot care (%)	100 (5)	37 (4)	63 (5)
Vitals/Exam (%)	99 (5)	48 (6)	51 (4)

No missing data. A complaint was either mentioned or was considered absent.

*ED*, emergency department; *MSK*, musculoskeletal; *PCP*, primary care physician.

**Table 4:** Odds ratios for people experiencing homelessness indicating they would have gone to the emergency department (ED) versus other location by demographics in the subset of Loyola Street Medicine’s patients with unique identifiers between October 2022 and September 2024 in a study examining the intersection of street medicine and the ED.

Demographics	Odds Ratio of ED (95% CI)	*P* Value
Age, per unit increase	1.00 (0.98–1.01)	.46
Male vs Female	1.14 (0.84–1.55)	.39
Race		
White (Reference)		
Black vs White	0.69 (0.49–0.97)	.03
Other vs White	0.39 (0.22–0.69)	.001
Hispanic vs non-Hispanic	0.70 (0.42–1.17)	.17
Insurance		
Medicaid (Reference)		
Private vs Medicaid	1.06 (0.74–1.52)	.73
None vs Medicare	0.91 (0.70–1.23)	.52
Other (Yes but unknown, Medicare, VA, Unsure / Unknown) vs Medicaid	0.88 (0.63–1.21)	.42
Duration of Homelessness		
<1 year (Reference)		
>1–5 years	1.15 (0.84–1.57)	.40
> 5 years	1.07 (0.72–1.58)	.75
Not Homeless	0.82 (0.55–1.24)	.35
Other (Intermittent, unknown, homeless but unsure for. how long)	0.69 (0.49–0.97)	.03

*ED*, emergency department; *VA*, Veterans Affairs.

**Table 5:** Odds ratios for people experiencing homelessness indicating they would have gone to the emergency department (ED) versus other location by chief complaints in the subset of Loyola Street Medicine patients with unique identifiers between October 2022 and September 2024 in a study examining the intersection of street medicine and the ED.

Chief Complaint	Odds Ratio of ED (95% CI)	*P* value
Respiratory	1.00 (0.69–1.46)	.10
Musculoskeletal	1.02 (0.74–1.39)	.92
Trauma / Injury	1.02 (0.39–2.64)	.97
Medication refill	1.10 (0.74–1.64)	.64
Wound care	1.46 (1.07–1.99)	.02
Rash / Bug bites / Dermatology	1.00 (0.68–1.49)	.98
Wellness check	0.68 (0.46–1.00)	.05
ENT, URI, Allergy	1.00 (0.69–1.47)	.99
Abdominal pain / GI	1.21 (0.58–2.56)	.61
Dental pain	1.10 (0.48–2.51)	.83
Vaccines	0.28 (0.05–1.71)	.17
Psychiatric	0.28 (0.05–1.62)	.16
Neurology	1.47 (0.84–2.55)	.18
Podiatry	0.80 (0.56–1.13)	.20
Ophthalmology	1.69 (0.65–4.41)	.28
Complaints related to substance use	1.03 (0.30–3.55)	.96
GU	1.45 (0.71–2.95)	.31
Heart / Chest pain	1.10 (0.41–2.95)	.85
Frostbite	0.35 (0.05–2.44)	.29
Other	0.82 (0.27–2.49)	.72
Number of Complaints		
0	Reference	
1	1.16 (0.49–2.76)	.75
2	1.21 (0.49–2.98)	.68
3 or 4	2.25 (0.64–7.99)	.21

*ED*, emergency department; *ENT*, ear, nose, and throat; *GI*, gastrointestinal; *GU*, genitourinary; *URI*, upper respiratory infection.

**Table 6:** Odds ratios for people experiencing homelessness indicating they would have gone to the emergency department (ED) versus other location by interventions in the subset of Loyola Street Medicine’s patients with unique identifiers between October 2022 and September 2024 in a study examining the intersection of street medicine and the ED.

Intervention	Odds Ratio of ED (95% CI)	*P* value
Medication Administered	1.06 (0.83–1.36)	.63
Type of medication administered		
Over the counter	0.962 (0.76–1.22)	.75
Inhaler	1.02 (0.73–1.43)	.90
Antibiotics or prescriptions medication	1.03 (0.85–1.66)	.30
Topicals	1.12 (0.82–1.53)	.46
Eye drops	1.46 (0.41–5.20)	.55
Pharmacy / prescription refill	1.16 (0.43–3.11)	.77
Other	1.43 (0.47–4.32)	.53
Vaccine Administered?	0.21 (0.02–1.38)	.21
Type of Vaccine		
Influenza	NE	
COVID-19	NE	
Wound care	1.50 (1.11–2.03)	.009
MSK intervention	0.75 (0.44–1.30)	.31
Referred to ED (or 911 call)	3.02 (1.35–6.74)	.007
Referred to social work	0.55 (0.21–1.49)	.24
Referred to PCP	0.90 (0.51–1.61)	.73
Foot Care	0.91 (0.55–1.51)	.72
Vitals/Exam	0.76 (0.45–1.27)	.30

*ED*, emergency department; *MSK*, musculoskeletal; *NE*, not estimable; *PCP*, primary care physician.
