# Supplementary material for: Impact of Street Medicine on Emergency Department Use: A Three-Year Evaluation of People Experiencing Homelessness
Source: West J Emerg Med. 2026 Sep 9;27(5):1457–66. doi: 10.5811/westjem.63074 (PMC13660663; doi:10.5811/westjem.63074)
Supplement: Supplementary file 1 [file wjem-27-1457-s001.docx]

**Figure 1.** Study flow diagram based on data collected between September 2021 and September 2024 in a study examining the intersection of street medicine and the ED.

*ED*, emergency department
